# Impact of online live broadcasts on environmental destructive behavioral intention

**DOI:** 10.1371/journal.pone.0286967

**Published:** 2023-06-13

**Authors:** Fengjun Xiao, Mengqian Xu, Jian Wu, Changsheng Meng, Yuxiang Hong

**Affiliations:** 1 Hangzhou Dianzi University, Hangzhou, China; 2 School of Politics and Public Administration, Guangxi Normal University, Guangxi, China; 3 School of Management, Hangzhou Dianzi University, Hangzhou, China; The Hong Kong Polytechnic University, HONG KONG

## Abstract

As information and communication technology advances rapidly, real-time live online broadcasting has emerged as a novel social media platform. In particular, live online broadcasts have gained widespread popularity among audiences. However, this process can cause environmental problems. When audiences imitate live content and perform similar field activities, it can have a negative effect on the environment. In this study, an extended theory of planned behavior (TPB) was used to explore how online live broadcasts relate to environmental damage from the perspective of human behavior. A total of 603 valid responses were collected from a questionnaire survey, and a regression analysis was conducted to verify the hypotheses. The findings showed that the TPB can be applied to account for the formation mechanism of behavioral intention of field activities caused by online live broadcasts. The mediating effect of imitation was verified using the above relationship. These findings are expected to provide a practical reference for the control of online live broadcast content and guidance on public environmental behavior.

## Introduction

As the foundation of human survival and development, the ecological environment is closely associated with a sustainable economic and societal development. In recent years, because of the rapid economic development in many countries, energy consumption has increased substantially [1]. Consequently, the ecological environment continues to deteriorate [2–4], posing a severe threat to the sustainable development of human society. In this context, a consensus has been reached in the international community to enhance environmental protection, preserve the ecological environment, and promote a sustainable development. As the Chinese economy has entered a new stage, environmental protection has also ushered in a period of important strategic opportunities. In its 13th Five-Year Plan, the Chinese government incorporated environmental protection as a basic policy in its national development plan to promote the protection and construction of ecological environments. Among various problems related to environmental protection, secondary environmental issues, which refer to the issues caused by human activities to the surrounding environment, have become an important part that must be considered for environmental protection and governance [5]. In general, human activities can be divided into two categories: industrial and daily production activities [6]. The regulation of industrial production activities relies mainly on various national policies such as upgrading the industrial system of factories, prohibiting hazardous production waste from being discharged before the standards are complied with, and paying attention to the protection of the original natural ecology when water-intensive factories and trunk roads are constructed [7]. Thus, the side effects of industrial production activities on the environment can be effectively minimized. Government policies can play a vital role in environmental protection, and significant contributions have been made by the Chinese government to the vigorous promotion of environmental protection. For the environmental protection practiced in daily production activities, attention should be paid to cultivating awareness of environmental protection among residents. They should be guided to start with small things, such as reducing the use of disposable and plastic products and avoiding littering [8]. However, this seemingly simple act can be difficult to perform. For the public, environmental awareness mostly comes from school education and everyday environmental protection propaganda such as slogans, televised advertisements promoting environmental protection, and public environmental protection activities on the Internet [9]. Because people live in an atmosphere full of environmental protection consciousness, they are subtly influenced by creating a social atmosphere of environmental protection for all people [10]. However, complex and fragmented information sources in modern society will inevitably have negative effects when the dissemination of environmental protection information is facilitated.

The Internet has exerted a strong influence as the most essential channel for information dissemination. Online video live broadcasting represents a novel method of information dissemination through which people can watch live videos online, such as games, conferences, teaching, and surgery, and it can intuitively and interestingly show what people intend to express with great viewing ability. It also enables real-time interaction between the video producer and audience, which is one of its social attributes [11]. Therefore, live broadcasts have become a form of online entertainment favored by current online user groups. By March 2020, the number of netizens in China reached 904 million, of which 560 million watched live broadcasts, accounting for as much as 61.95% of all Chinese netizens. Currently, people can gather interesting information quickly by watching live broadcasts of others to meet their entertainment and social needs [12, 13]. In addition, they can produce their own live broadcast content. Toffler described these types of users as “prosumers” [14]. Audiences can imitate or recreate the live content they are interested in without any restrictions and then post it on their personal social accounts. In addition to entertainment, uploaded videos can also be used to increase the attention of video uploaders to social platforms. Thus, popular content can be quickly disseminated across network communities.

While bringing entertainment content to people in their daily lives, broadcasts present opportunities and challenges to the development of society. For example, live broadcasts promote economic growth. Rural residents can rely on live broadcasts to sell local specialties or showcase the beautiful scenery of the countryside to attract tourists [15, 16]. Retailers can display the use and outcomes of their products on live broadcast platforms [17]. Outdoor travel bloggers can demonstrate their wilderness skills in real time while promoting the corresponding product [18]. However, in addition to creating economic benefits, live broadcasts also pose potential social risks. To increase attention and boost video playback volume, some bloggers introduce non-compliant or curious content into live broadcasts. For example, a blogger used to broadcast live cooking of Tianshan Saussurea, a wild plant under national first-level protection in the Tianshan Scenic Reserve, sparking public debate about it. In addition, some anchors travel to remote mountainous areas for expeditions without taking any safety measures. Eventually, social resources will be used to rescue them. In addition, human activities may have various negative effects, exacerbating the occurrence of ecological disasters [19, 20]. With the emergence of Internet celebrity attractions and live broadcast destinations, there has been a sharp rise in the number of tourists in the original remote and quiet field attractions. However, the quality of tourists is uneven, which causes tremendous pressure and potential safety hazards to local environmental protection efforts. For example, the act of making a fire in the wild may cause grassland or even forest fires. However, to compete for interest and traffic resources, some live video platforms recommend this content to audiences.

In addition, video producers habitually elaborate on how to achieve the same display effects. Live broadcasts enable audiences to send messages, raise questions, or share personal experiences. In addition to stimulating the interest of live broadcast audiences, this also reduces the level of difficulty for audiences to reproduce live content, especially when it comes to content that audiences usually lack access to, such as field activities. A representative of contemporary Internet pop culture is the spread and imitation of content of personal interest. Therefore, when the live content of field activities arouses the audience’s interest and conditions permit, some members of the audience tend to imitate the content in the form of live broadcasts, such as tree climbing and fishing. According to data collected from news reports, this phenomenon is currently on an upward trend. Because the main content of field activities is the interaction between humans and nature, human behavior inevitably affects the original natural environment during this process [21]. Dull content is usually ineffective in attracting audiences. Therefore, to enhance interest in video content, some bloggers choose to induce a certain degree of environmental damage in their live broadcasts. However, if a large audience imitates the content in a video by performing it in the field, even though the one-off destruction is far from severe, the destructive effect on the environment will be expanded by the superimposition of the same behavior.

As a significant component of sustainable development, environmental protection requires the participation of a large number of citizens to achieve better outcomes. More attention should be paid to social factors that may affect people’s awareness of environmental protection. Although there are many studies on the influence of media on individual’s environmental behaviors, there are few researches on the influence of “we media”, especially those focusing on the characteristics of audience’s imitative behaviors easily stimulated by exposure to online live broadcast of field activities. Therefore, in this study, the formation mechanism of the behavioral intention of individuals toward field activities was explained using an extended theory of planned behavior (TPB) model that considered the mediating effects of imitation in the context of online live broadcasts. The remainder of this paper is organized as follows. Section 2 presents the hypotheses based on a literature review. Section 3 presents the methodology, which involves the questionnaire design, ethical considerations, participants, and data analysis. Section 4 presents the results. In Section 5, a discussion is presented, and the conclusions are drawn in Section 6.

## From literature review to hypotheses

### Theory of planned behavior

**Fig 1:**
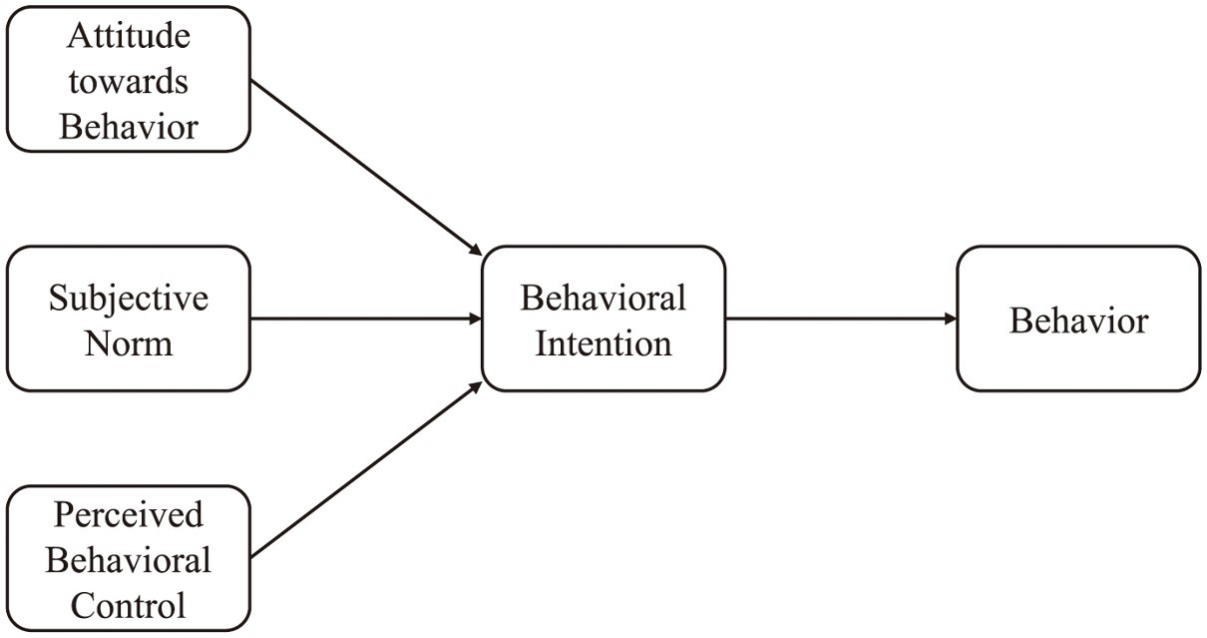
Framework for theory of planned behavior.

As one of the most widely used behavioral theories, the TPB was proposed by Ajzen in 1985. It is also one of the most popular theories for analyzing individual intentions and behaviors [22] and is applicable for analyzing the behavioral intention or motivation of users [23–25]. Under the TPB framework, an individual’s behavioral intention is determined by the attitude towards behavior, subjective norms, and perceived behavioral control [26], as shown in Fig 1.

In general, when an individual’s attitude towards a certain behavior tends to be positive, the behavioral intention is stronger. Similarly, when the subjective norms of a certain behavior tend to be positive, an individual’s behavioral intentions are reinforced. Subjective norms refer to the pressure from society that a person perceives when performing a certain activity. When an individual judges that he/she has sufficient resources and opportunities to do something while encountering few obstacles, the perceived behavioral control of this person will be stronger because of past experience and behavior. The TPB has been widely applied to predict the behavioral intentions of people toward field activities, including outdoor sports [27, 28], fishing [29, 30], and mountain climbing [31, 32]. TPB is also regarded as a powerful model for explaining environmental awareness [26, 33, 34], environmental exploration behavior [35], and environmental performance impact [36]. Based on the above discussion, the following hypotheses are proposed:

**Hypothesis 1 (H1): The attitude towards field activities has a positive impact on individual’s behavioral intention of field activities.**
**Hypothesis 2 (H2): Subjective norms exert a positive influence on individual’s behavioral intention of field activities.**
**Hypothesis 3 (H3): Perceived behavioral control has a positive impact on individual’s behavioral intention of field activities.**

### The mediating effect of imitation

How does the imitation behavior of netizens spread and evolve? The memetics (meme) theory proposed by Dawkins in “The Selfish Gene” can be used to answer this question [37]. Memes are cultural fragments that users copy and spread by imitating other netizens online. Internet memes include popular words, pictures, and small videos, all of which can be disseminated over a short period and have a substantial impact on social and cultural life [38]. The “lce Bucket Challenge” initiated in August 2014 serves as a typical example. This topic has been read more than 3.62 billion times on Weibo, a mainstream Chinese social platform similar to Twitter, and discussed more than 3.381 million times in as little as one week. Under the influence of this trend, many netizens spontaneously imitated it by participating in activities. Within less than half a month, more than 100,000 people uploaded videos that recorded the process of taking on the “lce Bucket Challenge” by themselves. In addition, the multichannel dissemination of topical videos such as “finger dance” and “seaweed dance” has contributed to the fission copying of content online. These are typical examples of network membranes.

**Fig 2:**
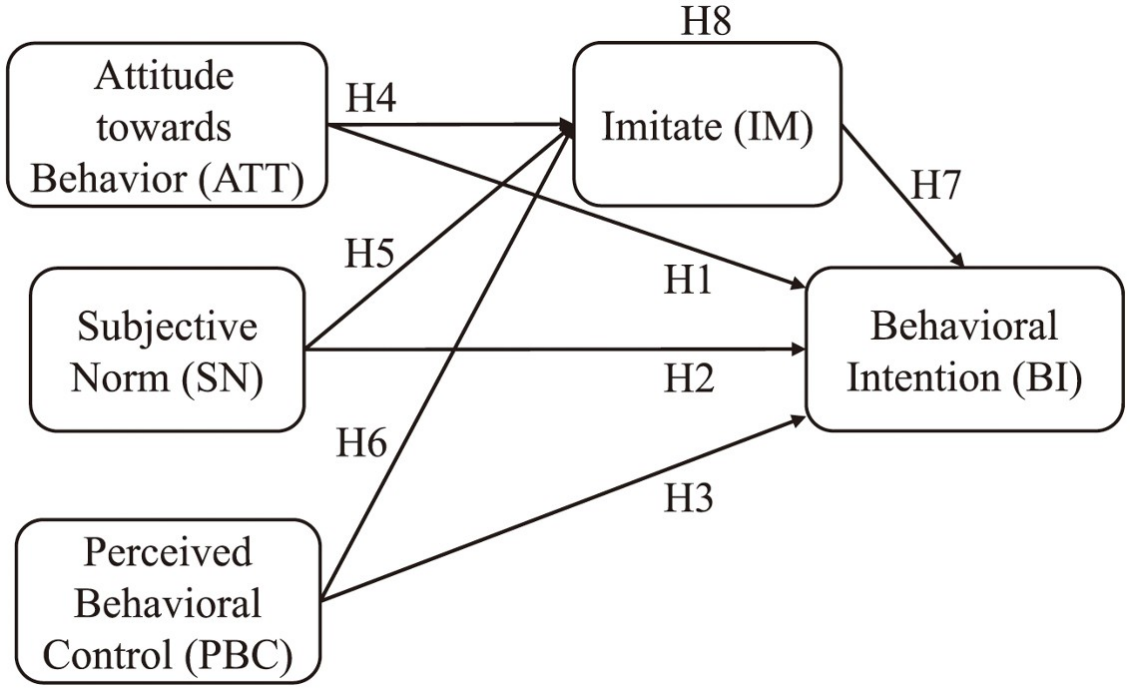
Theoretical framework of the mediator model.

Meme theory reveals that things are spread by network users through mutual imitation under the influence of Internet popularity. As a mode of learning, imitation involves the observation of others. In comparison, social learning involves learning the entire environment by observing others [39]. Imitation plays a vital role in social interactions [40, 41]. French sociologist Tarde described it in the book “Law of Imitation” as follows. Imitation is the most basic social relationship, and a society is a group of individuals who imitate each other [42]. Based on this, the following hypotheses are proposed. The relationship between the hypotheses and personal behavior is shown in Fig 2.

**Hypothesis 4 (H4): Attitude towards field activities has a positive impact on individual’s imitation.**

People usually support and share things they love or are interested in by spreading them through social processes. In contrast, they show disgust towards things they reject. Therefore, whether the user is interested in live broadcast content and supports the opinions expressed in this content largely determines whether the user intends to imitate it.

**Hypothesis 5 (H5): Subjective norms have a positive impact on individual’s imitation.**

Imitation is a common behavior in human society. When people have a positive attitude towards doing one thing, their personal attitude will be affected to the extent that they follow suit. Similarly, when the people around reject something, an individual’s willingness to act is suppressed. Therefore, to judge an individual’s willingness to imitate something, the surrounding social atmosphere must be considered.

**Hypothesis 6 (H6): Perceived behavioral control has a positive impact on individual’s imitation.**

Whether an individual imitates something is closely related to the individual’s life experiences. An individual tends to show a relatively strong willingness to take action when he/she judges that it is less difficult or acceptable in terms of cost, based on his/her knowledge reserve and past life experience. If an individual encounters great difficulty, the willingness to act is reduced. Considering that different people behave differently in terms of perceptual behavioral control when deciding whether to imitate others, a unified judgment standard would cease to be applicable.

According to meme theory, memes can be copied through imitation. Therefore, imitation is effective in expanding the cultural influence of social media and increasing the degree of attention paid to social events. For emerging Internet memes, netizens usually show a strong desire to imitate them and spontaneously forward, copy, and recreate them on social networks, making Internet memes rapidly popular [43, 44]. The stronger the netizens’ willingness to imitate, the higher is the possibility that they will imitate. Therefore, it can be concluded that the motivational intention of imitation reflects the probability of imitation. At the same time, under the influence of imitation motivation, there will be a change in personal behavior determined by the user’s attitude towards live broadcast, subjective norms, and perceived behavioral control. Therefore, this study suggests that imitation plays an intermediary role in the process of watching live broadcast content and conducting field activities. Therefore, the following hypotheses are proposed:

**Hypothesis 7 (H7): The imitation of live content of field activities has a positive impact on an individual’s behavioral intention toward field activities.**

In the TPB-based behavioral decision-making process, factors such as attitudes towards field activities, subjective norms, and perceived behavioral control affect an individual’s behavioral intention towards field activities. However, the external information with which the individual comes into contact, such as live videos, will have an impact on the effects of the above three factors on behavioral intention to some extent. This influence is treated as a mediating effect of the decision-making process.

**Hypothesis 8 (H8): Imitation plays a mediating effect in the TPB-based behavioral decision-making process.**
**Hypothesis 8 (H8a): Imitation mediates the relationship between attitude and behavioral intention.**
**Hypothesis 8 (H8b): Imitation mediates the relationship between subjective norms and behavioral intention.**
**Hypothesis 8 (H8c): Imitation mediates the relationship between perceived behavioral control and behavioral intention.**

## Methods

### Research design

We conducted a cross-sectional survey to validate the theoretical model. Before answering the questions, the participants were required to read a hypothetical scenario describing field-activity-related behavioral decision-making. Adopting the hypothetical scenario approach not only improves the reliability and validity of the measurement but also attracts the participants’ attention effectively [45]. After reviewing common live-broadcast content, the three most common scenarios were selected: digging bamboo shoots in the wild, fishing in the wild, and off-roading in the desert. Of these three scenarios, digging bamboo shoots in the wild is an activity that does not require special skills. Therefore, it is relatively easy to implement, and both individuals and groups can participate. Fishing in the wild requires a certain level of skill and usually involves the preparation and cooperation of multiple people. Off-roading in the desert places high requirements on participants. One must be proficient in driving, and the time required to reach the off-road field is another key influencing factor. By analyzing these three types of field activities with different difficulty levels, it is easy to cover and approach real scenarios.

### Measures

To achieve the aims of this research, a questionnaire was administered to collect data. Each measurement variable of the TPB as constructed in this study was either selected or adapted from Ajzen [46], whereas the measurement of imitation was self-developed. The items used to measure these five constructs required the use of a Likert scale [47] (strongly disagree = 1, disagree = 2, neutral = 3, agree = 4, fully agree = 5).

First, relevant professionals were invited to translate the scales previously published in authoritative journals. In October 2020, prior to the formal survey, expert interviews were conducted to verify the reliability of the scales in the context of China and online live broadcasting. The interviews were conducted with five participants, including two professors and two researchers who were familiar with this field and a work director with 10 years of work experience on a well-known live broadcast platform. Considering their suggestions, the original questionnaire was improved by adding and deleting details. Additionally, the wording of the questionnaire was refined to produce a simple and easy-to-understand measurement tool. The final questionnaire used for data collection is presented in Table 1.

**Table 1:** Measurement scales in the formal questionnaire.

Constructs and Measuring Items	Sources
Attitudes towards field activities (ATT)	[46]
ATT1: I think field activities are very interesting.	
ATT2: I think field activities are very fashionable.	
Subjective norms (SN)	[46]
SN1: People around have their favorite field activities.	
SN2: Relatives and friends are all interested in field activities.	
SN3: Many policies promote and encourage going to the wild.	
Perceived behavioral control (PBC)	[46]
PBC1: I think I am very good at certain field activities.	
PBC2: I have plenty of time for field activities.	
PBC3: I have sufficient resources to conduct field activities.	
Imitation of live broadcast field activities (IM)	self-developed
IM1: If I really like the video anchors in the field activities, I will want to imitate the corresponding things.	
IM2: If the video of field activities is very popular, I would really like to imitate the corresponding actions.	
Willingness to participate in field activities (BI)	[46]
BI1: I have the intention of field activities	
BI2: I am very willing to engage in field activities.	

### Ethical considerations

This study was approved by the Ethics Committee of the Hangzhou Dianzi University. Participation in this study was based on obtaining informed consent. Each questionnaire was accompanied by a cover letter, information sheet, and consent form. All respondents were adults, and they were provided with complete written information about the study before the questions were answered.

### Participants

The questionnaire survey was conducted online by our research group through a questionnaire website for two reasons. On the one hand, our research mainly focuses on Internet users, and an online questionnaire is more consistent with the actual circumstances of the target user group. On the other hand, an online questionnaire can be sent more easily to the target user group as it can be distributed to users across the country in a low-cost and highly efficient manner. Such investigative methods are increasingly being used in relevant research fields [48–50].

The questionnaire was distributed through two channels from November 2020 to mid-January 2021. One was a general survey that sent a link to the questionnaire through social media (such as QQ and WeChat), randomly targeting online users aged between 18–50 years old across the country. Using snowball sampling, respondents were invited to forward the questionnaire to their friends, colleagues, classmates, and so on. Thus, it was ensured that the questionnaire was distributed efficiently and in large numbers. The other channel was a survey targeting a specific group, with the questionnaire sent to the audience of a live broadcast platform by six well-trained investigators. The purpose of completing the questionnaire and details of the concept of on-site seeding were communicated to potential interviewees.

Using the two distribution methods, 300 questionnaires were issued under each scenario (i.e., digging bamboo shoots in the wild, fishing in the wild, and off-roading in the desert), resulting in a total of 900 questionnaires. In total, 758 respondents completed the questionnaire (response rate: 84.2%). First, the data were filtered to ensure questionnaire validity. After excluding questionnaires with blank or incomplete answers or contradictory questions, 603 questionnaires were retained for analysis, including digging bamboo shoots in the wild (200), catching fish in the wild (210), and off-roading in the desert (193). According to the requirements of Kline [51], at least ten valid answer sheets were required for each question. Considering the three items studied, at least 30 valid responses were required. In our questionnaire feedback, 603 valid answers met this requirement. Statistical analyses were conducted on the composition and structure of the questionnaires (Table 2).

**Table 2:** Sample characteristics of 603 participants.

Demographic factors	Groups	Frequency	Percentage
Sex	Male	392	65%
	Female	211	35%
Age	Under 25	373	61.8%
	26–40	205	34.0%
	40 and over	25	4.2%
Education	Senior high school or below	59	9.8%
	Junior college	36	6.0%
	Bachelor’s degree	345	57.2%
	Master’s degree or above	163	27.0%

### Data analysis

In this study, the reliability and validity of the measurements were tested using a confirmatory factor analysis (CFA). A hierarchical regression analysis was conducted to verify the hypotheses. SPSS 19.0 and AMOS 17.0 were used to analyze the data.

## Result

### Reliability and validity testing

The Cronbach’s *α* coefficients of the willingness to participate in field activities (BI), attitudes towards field activities (ATT), subjective norms (SN), perceived behavioral control (PBC), and imitation of live broadcast field activities (IM) were 0.896, 0.845, 0.904, 0.909, and 0.878, respectively, indicating the high internal consistency of the questionnaire. According to the results, the KMO value was 0.938 > 0.8, and Bartlett’s test of sphericity was significant. In other words, the data had good structural validity and were suitable for factor analysis.

### Confirmatory factor analysis

Before data analysis, a CFA was conducted using AMOS17.0 to verify the discriminant validity of the variables in this study (see Table 3). All five variables were factored into a measurement model to create the latent constructs that freely covaried. The five-factor model fit the data well (*χ*^2^/*df* = 3.87, *TLI* = 0.971, *CFI* = 0.981, *RMSEA* = 0.069). When both AT and SN were restricted to being part of the same factor, the four-factor model fit decreased (*χ*^2^/*df* = 6.978, *TLI* = 0.940, *CFI* = 0.956, *RMSEA* = 0.10). Moreover, when both SN and PBC were restricted from being part of the same factor, the four-factor model fit decreased (*χ*^2^/*df* = 9.743, *TLI* = 0.912, *CFI* = 0.936, *RMSEA* = 0.121). Subsequently, three-factor and two-factor models were constructed, and the degree of fitting gradually decreased. Finally, a one-factor model was constructed that included all five variables and the model fit was reduced (*χ*^2^/*df* = 21.396, *TLI* = 0.794, *CFI* = 0.831, *RMSEA* = 0.184). In summary, the five variables have good discriminant validity, representing five different constructs.

**Table 3:** Results of confirmatory factor analysis.

Measurement Model with Variables	*χ*^2^/*df*	TLI	CFI	RMSEA
five-factor model: BI;ATT;SN;PBC;IM	3.87	0.971	0.981	0.069
four-factor model: BI;ATT;SN+PBC;IM	6.978	0.940	0.956	0.10
four-factor model: BI;ATT+SN;PBC;IM	9.743	0.912	0.936	0.121
three-factor model: BI;ATT+SN+PBC;IM	13.773	0.871	0.900	0.146
two-factor model: BI;ATT+SN+PBC+IM	17.685	0.831	0.865	0.166
one-factor model: BI+ATT+SN+PBC+IM	21.396	0.794	0.831	0.184

### Preliminary results

Table 4 presents the means, standard deviations, and correlation coefficients of the variables. All five variables (BI, ATT, SN, PBC, and IM) were positively correlated. These results supported the hypotheses proposed in this study.

**Table 4:** Means, standard deviations, and correlation coefficient.

Variables	Means	SD	BI	ATT	SN	PBC
BI	2.953	0.931				
ATT	3.179	0.880	0.733			
SN	2.829	0.875	0.692	0.681		
PBC	2.644	0.930	0.638	0.587	0.826	
IM	3.022	0.894	0.734	0.728	0.732	0.638

### Primary analyses

As presented in Table 5, regression models were constructed for the BI (Models 1–3) and IM (Models 4–5) variables. A regression model was established using control variables (Model 1). The three variables of the TPB were then factored into Model 2. According to the results, ATT (Model 2, *β* = 0.523, *ρ* < 0.001), SN (Model 2, *β* = 0.231, *ρ* < 0.001), and PBC (Model 2, *β* = 0.160, *ρ* < 0.01) exerted a positive influence on intentions. Thus, hypotheses 1–3 were supported. Imitation was then entered into Model 3 with the results showing that IM had a positive effect on BI (Model 3, *β* = 0.318, *ρ* < 0.001). Thus, Hypothesis 7 was supported. Meanwhile, the influencing coefficients decreased for the ATT, SN, and PBC that impacted BI, indicating a mediating effect of IM. Subsequently, a regression model was constructed for IM. It was found out that both ATT (Model 5, *β* = 0.458, *ρ* < 0.001) and SN (Model 5, *β* = 0.376, *ρ* < 0.001) had positive impacts on IM, whereas the effect of PBC on IM was insignificant. Thus, hypotheses 4–5 were supported, whereas hypotheses 6 and 8c were rejected.

**Table 5:** Results of regression.

	BI						IM			
	Model 1		Model 2		Model 3		Model 4		Model 5	
	*β*	*ρ*	*β*	*ρ*	*β*	*ρ*	*β*	*ρ*	*β*	*ρ*
GE	-0.170	0.039	-0.057	0.291	-0.044	0.390	-0.143	0.068	-0.040	0.430
AG	0.057	0.179	-0.025	0.348	-0.023	0.378	0.064	0.114	-0.008	0.749
EDU	-0.110	0.006	-0.021	0.410	-0.006	0.821	-0.141	<0.001	-0.049	0.041
SCE	-0.075	0.139	-0.019	0.553	-0.032	0.297	-0.013	0.791	0.041	0.172
ATT			0.523	<0.001	0.377	<0.001			0.458	<.001
SN			0.231	<0.001	0.112	0.037			0.376	<0.001
PBC			0.160	0.001	0.144	0.001			0.053	0.226
IM					0.318	<0.001				
R2	0.028		0.618		0.651		0.034		0.640	
F	4.228		137.419		138.770		5.301		151.278	
ΔR2	0.028		0.590		0.034		0.034		0.606	
ΔF	4.228		306.370		57.265		5.301		334.104	

To verify the mediating effects of IM on the relationship between attitudes and BI, we follow the testing procedure specified by Zhao et al [52]. Using the PROCESS provided by Hayes [53], we estimated 5000 bootstrap samples in which the independent variable was ATT, the mediator was IM, and the dependent variable was BI. Gender (GE), age (AG), education (EDU), and scenario (SCE) were also included as covariates in the model. The results showed that IM partially mediated the relationship between attitude and BI (direct effect = 0.473; 95% confidence interval (0.3942, 0.5526); indirect effect = 0.319; 95% confidence interval (0.2306, 0.4130)). Therefore, Hypothesis 8a was supported. The same method was used to test the mediating effects of IM on the relationship between SN and BI. The results showed that IM partially mediates the relationship between SN and BI (direct effect = 0.363; 95% confidence interval (0.2820, 0.4438); indirect effect = 0.376; 95% confidence interval (0.2912, 0.4601)). Therefore, Hypothesis 8b was supported.

## Discussion

The research conducted in this study shows that imitation motivation exerts a mediating effect on the field activity behavior of users regarding content in live broadcasts, especially their attitudes and subjective norms, while the impact on perceived behavioral control is relatively limited. User’s attitudes towards field activities and subjective norms are consistent with the conclusions drawn in other studies [54, 55]. The results also show that personal attitudes and subjective norms play an essential role in determining whether live broadcast audiences engage in field activities. Simultaneously, the influence of imitation motivation on these two personal factors will increase or decrease the probability of imitating wild activities, which hinges on the interest of audiences in the live content they see and the degree of recognition given to the content. However, the effect of imitation behavior on perceived behavioral control is relatively limited. Thus, Hypothesis 6 was rejected. Many studies have shown that the perceived behavioral control exerts a positive effect on personal behavioral intentions, such as green shopping and the use of energy-saving appliances [56, 57]. However, only a few studies have obtained results similar to those of the present study. It is speculated that this is a result of the living conditions in Chinese society. A large proportion of the groups watching live broadcasts are young office workers and students who usually have a stronger interest in field activities. However, performing field activities requires not only certain skills such as catching fish and driving, but also a proportionate amount of free time. Currently, students usually come under high learning pressure, which restricts them to spend only fragmented time watching live broadcasts or performing physical exercises, whereas young working people are more inclined to spend their free time staying at home or engaging in light entertainment with friends. For the main audience groups of live broadcasts, spending a significant amount of time in wildlife areas for unskilled field activities may be a desirable form of entertainment and leisure. However, this may necessitate considerable preparation work, such as learning to drive and use fishing gear, both of which are not priority activities for most people in today’s fast-paced social lives. Therefore, imitation behavior does not make any considerable difference to the perceptual behavioral control of the live audience group.

Motivation for imitation can influence personal attitudes to a certain degree. According to the theory of the French sociologist Tarde proposed in the book “The Law of Imitation,” people tend to imitate those people or behaviors that they feel have a higher social hierarchy. Simultaneously, without interference, the imitation grows exponentially and rapidly spreads once it begins [42]. An individual’s attitude towards field activities is usually determined by the relevant education received [58, 59], such as preserving the environment and not lighting fires in the wild. However, in the absence of supervision, whether people perform relevant behaviors relies only on personal self-discipline. With the advancement of compulsory education and the popularization of Internet, most young netizens now have a strong awareness of the law. In addition, they try to avoid illegal behavior when performing field activities. Therefore, despite the possibility of some novel outdoor content in the live broadcast stimulating the interest of the live broadcast audience, the audience group will have a certain recognition ability and will not blindly follow this content before considering whether the behavior in the live broadcast complies with the regulations and whether they can do it. However, some popular clips, such as singing and dancing on the Internet, are easy to imitate and continue to spread without affecting the surrounding people or the environment. This is because the live broadcast audience will judge whether the content is compliant according to the actual situation. Therefore, when live broadcasts related to wild activities are conducted by video bloggers, it is best to avoid broadcasting content that will be detrimental to the wild environment because this may trigger psychological resistance among the audience. By broadcasting content related to environmentally friendly behaviors, the dissemination of video content can be facilitated and made more consistent with the current mainstream values of society, as people and nature should live in harmony, thus leaving a positive impression on the minds of the audience.

Personal subjective norms affect engagement in field activities, and imitation motivation exerts an intermediary effect during this process. In a multiperson participation activity, when most people participate, the few individuals who do not usually feel pressured. When a live broadcast is watched, if the surrounding people imitate the content shown in the live broadcast, the individual usually follows this general trend to avoid becoming an alternative individual [60]. Owing to the influence of external groups, the concepts and behaviors of most people tend to be the same. This is known as group psychology for various social behaviors and events [61]. Therefore, when a group of people is active in the wild, and most of them intend to imitate the content of the live broadcast they watch, it is easy to spread this imitation behavior across the group. When the content of this activity damages the environment, the increase in the number of people participating in the activity exacerbates the damage caused to the environment. When most people in a group refuse to imitate behaviors that are damaging to the environment, the possible imitating behaviors are suppressed, thus producing a protective effect on the environment. Therefore, under the influence of imitation motivation, personal subjective norms can exert both positive and negative effects on the environment, depending on the specific content of the field activities and the attitude of most people in the group towards the content of field activities.

## Conclusions

This study explored the environmental impact of the imitation behavior of users of live broadcast content in the Chinese context. In this study, aside from personal attitudes, subjective norms, and perceived behavioral control, the mediation factor of imitating motivation was mainly explored when acting on the above factors, which have an impact on individual behavior. The results indicate that the proposed model is robust and produces predictive effects. The intention of live broadcast audiences to imitate the content of a live broadcast in the wild can be analyzed and predicted through personal attitudes, subjective norms, perceived behavior, and imitation motivation. Personal attitudes and subjective norms play a major role. The influence of imitation motivation on these two aspects is more likely to further affect personal behavior, whereas the influence of perceived behavioral control in this process is less significant. Moreover, imitation motivation is less likely to influence it.

This work has some limitations that need to be addressed in future studies. First, the sample size was insufficient to meet the specified requirements. Therefore, in future research, it will be necessary to increase the number of interviewees and their number in different categories to better reflect the impact of live broadcast content on different groups. In addition, the different groups tended to show different degrees of preference for different live content types. When viewers have little or no live broadcast content, it is difficult for them to decide whether to adopt imitating behavior after watching the corresponding content. Second, according to the results of the questionnaire survey, there was a distinction between groups living in cities and those living in rural areas with respect to imitation of wild activities. This is related to whether they are more likely to be exposed to wild environments [62]. At the same time, the average level of education and legal concepts perceived by urban residents were slightly higher than those perceived by rural residents. We speculate that a significant difference is caused by these two factors combined. Therefore, in future research, questionnaire surveys should be conducted separately for these two groups. Third, the willingness to imitate behavior in live broadcasts was measured in this study; however, this does not mean that imitating behavior will necessarily be implemented. With the improvement in environmental protection awareness among people and the tightening of supervision of live broadcast platform content, behaviors that have a detrimental effect on the environment will gradually disappear from people’s lives to further cultivate the public’s responsibility and awareness of environmental protection. As the development of live broadcasts in China is at its peak, the content obtained by netizens and viewers from live broadcasts is diverse and complex. In addition, young people are more likely to be influenced by popular information [63] and imitate the people or things seen in live broadcasts.

Therefore, it is necessary to assess the social impact of the content of live-streaming platforms. For content that may have negative impacts, such as that from imitators promoting destructive actions on the environment, disseminators should be encouraged to embed reminders of environmental protection-related issues in their content. Measures such as tightening the supervision of live broadcast platforms and formulating detailed specifications, as well as encouraging anchors to produce more positive guiding videos, can contribute to the promotion of ecological environmental protection, which is conducive to increasing the awareness of ecological environmental protection in the entire population on a continuous basis.

## Supporting information

S1 Data (SAV)
